# Na/H Exchange Regulatory Factor 1 Deficient Mice Show Evidence of Oxidative Stress and Altered Cisplatin Pharmacokinetics

**DOI:** 10.3390/antiox10071036

**Published:** 2021-06-28

**Authors:** Adrienne M. Bushau-Sprinkle, Michelle T. Barati, Yuxuan Zheng, Walter H. Watson, Kenneth B. Gagnon, Syed Jalal Khundmiri, Kathleen T. Kitterman, Barbara J. Clark, Leah J. Siskind, Mark A. Doll, Michael E. Brier, Susan Coventry, Eleanor D. Lederer

**Affiliations:** 1 Department of Pharmacology and Toxicology, University of Louisville, Louisville, KY 40202, USA; ambush03@louisville.edu (A.M.B.-S.); yuxuan.zheng@louisville.edu (Y.Z.); bert.watson@louisville.edu (W.H.W.); leah.siskind@louisville.edu (L.J.S.); mark.doll@louisville.edu (M.A.D.); michael.brier@louisville.edu (M.E.B.); 2 Department of Medicine, Division of Nephrology, University of Louisville, Louisville, KY 40202, USA; michelle.barati@louisville.edu (M.T.B.); kathleen.kitterman@louisville.edu (K.T.K.); 3 Department of Medicine, Division of Gastroenterology, Hepatology and Nutrition, University of Louisville, Louisville, KY 40202, USA; 4 Division of Nephrology and Charles and Jane Pak Center for Mineral Metabolism and Clinical Research, University of Texas Southwestern Medical Center, Department of Medicine, Dallas, TX 75390, USA; Kenneth.Gagnon@UTSouthwestern.edu; 5 Department of Physiology and Biophysics, College of Medicine, Howard University, Washington, DC 20059, USA; syed.khundmiri@Howard.edu; 6 Department of Biochemistry and Molecular Genetics, University of Louisville, Louisville, KY 40202, USA; barbara.clark@louisville.edu; 7 Department of Pathology, University of Louisville, Louisville, KY 40202, USA; susan.coventry@nortonhealthcare.org; 8 Department of Pediatrics, University of Louisville, Louisville, KY 40202, USA; 9 VA North Texas Health Sciences Center, Dallas, TX 75216, USA

**Keywords:** cisplatin nephrotoxicity, redox status, acute kidney injury

## Abstract

(1) Background: One third of patients who receive cisplatin develop an acute kidney injury. We previously demonstrated the Na/H Exchange Regulatory Factor 1 (NHERF1) loss resulted in increased kidney enzyme activity of the pentose phosphate pathway and was associated with more severe cisplatin nephrotoxicity. We hypothesized that changes in proximal tubule biochemical pathways associated with NHERF1 loss alters renal metabolism of cisplatin or response to cisplatin, resulting in exacerbated nephrotoxicity. (2) Methods: 2–4 month-old male wild-type and NHERF1 knock out littermate mice were treated with either vehicle or cisplatin (20 mg/kg dose IP), with samples taken at either 4, 24, or 72 h. Kidney injury was determined by urinary neutrophil gelatinase-associated lipocalin and histology. Glutathione metabolites were measured by HPLC and genes involved in glutathione synthesis were measured by qPCR. Kidney handling of cisplatin was assessed by a kidney cortex measurement of γ-glutamyl transferase activity, Western blot for γ-glutamyl transferase and cysteine S-conjugate beta lyase, and ICP-MS for platinum content. (3) Results: At 24 h knock out kidneys show evidence of greater tubular injury after cisplatin and exhibit a decreased reduced/oxidized glutathione ratio under baseline conditions in comparison to wild-type. KO kidneys fail to show an increase in γ-glutamyl transferase activity and experience a more rapid decline in tissue platinum when compared to wild-type. (4) Conclusions: Knock out kidneys show evidence of greater oxidative stress than wild-type accompanied by a greater degree of early injury in response to cisplatin. NHERF1 loss has no effect on the initial accumulation of cisplatin in the kidney cortex but is associated with an altered redox status which may alter the activity of enzymes involved in cisplatin metabolism.

## 1. Introduction

**Figure 1:**
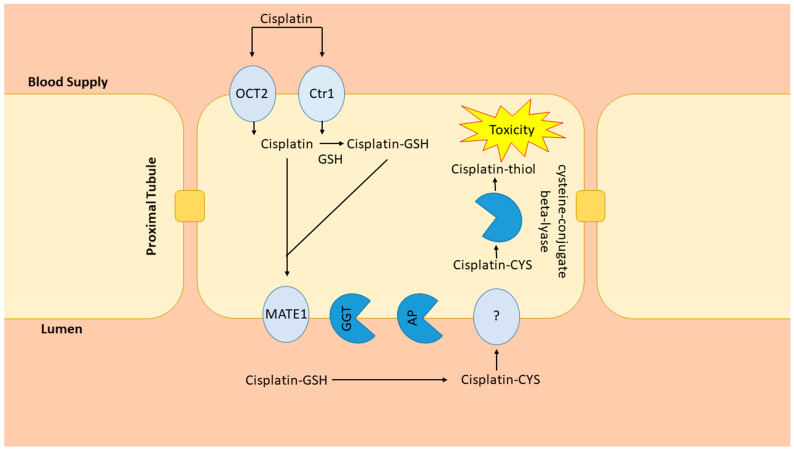
Renal cisplatin metabolism. Cisplatin uptake by OCT2 and Ctr1 is followed by conjugation to glutathione (GSH). MATE-1 secretes cisplatin and cisplatin-glutathione conjugates into the tubule lumen where they are metabolized by ƴ-glutamyl transpeptidase (GGT) and aminodipeptidase (AP) to cysteinyl and cysteine conjugates. The cisplatin-cysteine conjugate is taken up into the renal proximal tubule through unknown pathways and metabolized to an unstable and reactive cisplatin-thiol conjugate, the nephrotoxic metabolite of cisplatin, by cysteine beta-lyase.

Cisplatin, a highly efficacious chemotherapeutic agent, is used in the treatment of a wide variety of common malignancies [1,2,3]. Cisplatin crosslinks with purine bases in DNA and inhibits DNA synthesis, leading to cell death [2]. Because cisplatin shows its most potent cell toxicity in highly proliferative cells, the mechanism for its dose-dependent and cumulative nephrotoxicity remain largely unexplained [1,2,3]. Upwards of 20–30% of patients receiving cisplatin develop acute kidney injury (AKI) with a single dose [3]. The development of AKI essentially eliminates the use of this potent agent; therefore, determining why some individuals develop AKI remains an important area of research. Because the kidneys are the major site for cisplatin elimination and the target for injury, much research has been devoted to understanding how cisplatin is handled by the kidneys. Cisplatin accumulates in kidney proximal tubule cells through uptake by the organic ion transporter-2 (OCT2) and copper transporter-1 (Ctr1) located on the basolateral membrane [4,5] (Figure 1). On the apical membrane side, the multidrug and toxin extrusion protein-1 (MATE1) transports cisplatin from the proximal tubule to the urine [6] (Figure 1). Support for the contribution of these pathways comes from studies showing that OCT2 inhibition blunted cisplatin nephrotoxicity while MATE1 inhibition intensified toxicity in mice [5,6,7]. The kidney metabolism of cisplatin is complex, beginning with the formation of glutathione conjugates after proximal tubule uptake [8], followed by the cleavage to cysteinyl-glycine-conjugates by γ-glutamyl transferase (GGT), also expressed in proximal tubule cells [8] (Figure 1). 

From there, aminopeptidase (AP) converts cysteinyl-glycine-conjugates to cysteine conjugates, reabsorbed into the proximal tubule and converted into nephrotoxic thiols [8] (Figure 1). This schema, however, has not been firmly established as studies have resulted in contradictory findings. For example, because GGT has the highest activity in the kidney, logically it would seem to be an ideal target for the attenuation of nephrotoxicity. Yet, investigations into that possibility have yielded inconsistent results, with some studies demonstrating protective and some suggesting intensified nephrotoxicity when GGT activity is enhanced [9]. 

Our laboratory has identified a new potential target to ameliorate cisplatin nephrotoxicity, Na/H exchange regulatory factor isoform 1 (NHERF1), a scaffolding protein highly expressed in renal proximal tubule cells and critical for the regulation of solute transport and hormone receptor signaling pathways [10]. Previously we demonstrated NHERF1 knock out (KO) mice exhibited exacerbated cisplatin nephrotoxicity, including significantly greater increases in blood urea nitrogen (BUN), neutrophil gelatinase-associated lipocalin (NGAL), and histologic injury severity scores compared to the wild-type (WT) littermates [11]. NHERF1 KO mice showed similar levels of apoptosis but higher levels of necrosis when compared to WT mice [11]. Studies from other laboratories have demonstrated cisplatin nephrotoxicity dose dependence and higher levels of necrosis associated with higher concentrations of cisplatin, suggesting that the NHERF1 KO mouse kidneys may handle cisplatin differently than WT [12,13]. In previous studies, we demonstrated that kidneys from NHERF1 KO mice exhibit greater enzyme activity in the pentose phosphate pathway, suggesting that NHERF1 regulates biochemical pathways [14]. In addition to this, we have found in aging rats that there is a decrease in NHERF1 expression [15]. As aging is associated with changes in drug metabolism, these data support the suggestion that NHERF1 expression may influence proximal tubule drug handling. 

The goal of this work was to explore a novel mediator of susceptibility to cisplatin-induced AKI. We hypothesized that the effects of NHERF1 loss on kidney metabolic pathways affected the renal transport and/or metabolism of cisplatin. 

## 2. Materials and Methods

### 2.1. Animals and Treatments

2-4 month-old male WT C57BL/6J and C57BL/6J NHERF1(−/−) KO littermate mice [16] were housed in a pathogen-free barrier facility, maintained on a 12:12 h light–dark cycle and were provided water and food ad libitum. Animals were maintained on the same diet and for the same period of time to mitigate changes in cysteine levels. Body weights were recorded prior to cisplatin administration and at the time of sacrifice (Figure S1). All studies were performed at the same time each day to mitigate any fluctuations due to circadian rhythm that may also affect the study. At the time of sacrifice, animals were anesthetized with ketamine/xylazine (100/15 mg/kg, intraperitoneally (IP)). For the cisplatin studies, mice received a single IP injection of 20 mg/kg cisplatin (University of Louisville hospital pharmacy; Intas Pharmaceuticals Limited, Pharmez, Ahmedabad-382-213, India) or vehicle (saline). Vehicle treated and cisplatin treated mice were euthanized after 4, 24, or 72 h. Kidneys were removed and decapsulated before being: [1] immersed in 3.7% formaldehyde in phosphate buffered saline (PBS) for 24 h and then transferred to 70% ethanol prior to paraffin embedding for histology and IHC; [2] collected in ice-cold PBS with 1% penicillin/streptomycin for kidney cortex homogenization; or [3] snap frozen in liquid nitrogen and stored at −80 °C for further analysis. All animals used in these studies were in accordance with the guidelines established by the Institutional Animal Care Use Committee (IACUC) at the University of Louisville and followed the guidelines of the American Veterinary Medical Association.

### 2.2. γ-Glutamyl Transferase Activity Assay

The activity assay was performed using the GGT colorimetric assay kit (Sigma Aldrich; St. Louis, MI, USA) per the manufacturer’s instructions. Tissue samples were prepared using approximately 10 mg of kidney cortex in 200 μL of ice-cold GGT assay buffer. The samples were then centrifuged at 13,000× *g* for 10 min to remove insoluble material. The supernatant was kept and further diluted to 1:100 using the GGT assay buffer and stored at −80 °C until further use. 4 h: (WT vehicle: n = 6), (NHERF1 KO vehicle: n = 6), (WT cisplatin: n = 6), and (NHERF1 KO cisplatin: n = 6) and 24 h: (WT vehicle: n = 5), (NHERF1 KO vehicle: n = 5), (WT cisplatin: n = 7), and (NHERF1 KO cisplatin: n = 8).

### 2.3. Histology and Immunohistochemistry (IHC)

Paraffin embedded fixed whole kidney tissues were cut in 5 µm thick sections, either transversely or longitudinally from cisplatin treated and vehicle treated WT and NHERF1 KO animals. These sections were stained with hematoxylin and eosin (H&E) as well as periodic acid-schiff (PAS) and sent to a pathologist to evaluate the number of casts, degree of brush border membrane (BBM) sloughing, and acute tubular necrosis (ATN). Images were taken at 20× and 40× (WT vehicle n = 5), (NHERF1 KO vehicle n = 5), (WT cisplatin n = 7), and (NHERF1 KO cisplatin n = 8).

IHC was carried out on paraffin embedded fixed whole kidney tissue cut in 5 µm thick sections either transversely or longitudinally from cisplatin treated and vehicle treated WT and NHERF1 KO animals. A primary antibody against GGT (1:50) (ABclonal; Woburn, MA; A1776) and CCBL1 (ABclonal; A6542) was utilized for staining to evaluate any changes in proximal tubule localization or loss after cisplatin treatment. CCBL1 stained slides were blinded and further evaluated for uniform vs. patchy staining, granular staining, tubule mosaic staining, overall level of proximal tubule staining, and tubule injury. Images were taken at 40×. GGT: [(WT vehicle: 4), NHERF1 KO vehicle: 5), (WT cisplatin: 4), and (NHERF1 KO cisplatin 5)] and CCBL: [(WT vehicle: n = 5), (NHERF1 KO vehicle: n = 6), (WT cisplatin: n = 4), and (NHERF1 KO cisplatin: n = 5)]. 

### 2.4. Neutrophil Gelatinase-Associated Lipocalin (NGAL)

NGAL was determined using an enzyme linked immunosorbent assay (ELISA) kit (R&D Systems; Minneapolis, MN; DY1857, USA) on mouse urine as directed by the manufacturer (WT vehicle n = 5), (NHERF1 KO vehicle n = 5), (WT cisplatin n = 7), and (NHERF1 KO cisplatin n = 8). 

### 2.5. Collection of Mouse Blood and Tissue for Glutathione (GSH), Glutathione Disulfide (GSSG), Cysteine (Cys), and Cystine (CySS)

Blood samples were collected from mice via retro-orbital bleed and added to a tube with 0.5 M borate buffer stock solution and 165 uM γ-glutamylglutamate as the internal standard as described in [17] within 1–2 min after the blood was drawn. Plasma was extracted via centrifugation (3000× *g* for 2 min) and 50 µL of supernatant was transferred to a new tube with preservation solution (10% perchloric acid, 0.2 M boric acid, and 10 µM γ-glutamylglutamate). Tubes were then stored at −80 °C. In addition, 10 mg of kidney cortex tissue was removed from the kidney and added to the preservation solution (5% perchloric acid, 0.2 M boric acid, and 10 µM γ-glutamylglutamate) and homogenized. Kidney cortex homogenates were then stored at −80 °C until further use. 

### 2.6. HPLC Analysis of Kidney Cortex and Plasma for GSH, GSSG, Cys, and CySS 

Tissue and plasma Cys, CySS, GSH, and GSSG were assayed by HPLC as S-carboxymethyl, N-dansyl derivatives using γ-glutamylglutamate as an internal standard as previously described [17,18]. The HPLC system consisted of an aminopropyl column, 2 Waters 515 HPLC Pumps to deliver a gradient of acetate and methanol, a Waters 717 plus Autosampler with refrigeration unit, and a Waters 2475 Multi λ Fluorescence Detector. Integration of peak areas and comparison to the internal standard was performed by Waters Empower 3 software. Plasma: (WT vehicle: n = 5), (NHERF1 KO vehicle: n = 5), (WT cisplatin: n = 7), and (NHERF1 KO cisplatin: n = 9). Kidney: (WT vehicle: n = 5), (NHERF1 KO vehicle: n = 5), (WT cisplatin: n = 8), and (NHERF1 KO cisplatin: n = 9).

### 2.7. RNA Isolation and Quantitative Reverse-Transcriptase Polymerase Chain Reaction (PCR) 

Total RNA was isolated from the kidney cortex using an Ambion mirVana mRNA isolation kit per the manufacturer’s instructions (ThermoFisher, Austin, TX, USA). cDNA was synthesized from 1000 ng total RNA using SuperScript Vilo Master Mix (ThermoFisher) following the manufacturer’s instructions. Real-time quantitative PCR (qPCR) was conducted to measure Slc7a11, Abcc1, Gclc, Gclm, Abcc5, Gss, and GAPDH mRNA expression with TaqMan probes (TaqMan^®^ Gene Expression Assay Mm00442530_m1, Mm01344332_m1, Mm00802655_m1, Mm01324400_m1, Mm01343626_m1, Mm00515065_m1, Mm99999915_g1; Applied Biosystems), according to the manufacturer’s protocol (TaqMan Universal Master Mix II; Applied Biosystems; Waltham, MA, USA). Step One Plus Real Time PCR System (Applied Biosystems; Waltham, MA, USA) was used for qPCR with the parameters: 50 °C 2 min, 95 °C 10 min, followed by 40 cycles of 95 °C 15 s and 60 °C 1 min. Results were analyzed using Step One software version 2.3 (Applied Biosystems; Waltham, MA, USA). The amplification curves were analyzed by the mathematical equation of the second derivative, and the amounts of *Slc7a11*, *Abcc1*, and *Gclc* mRNA expression were normalized to the housekeeping gene *Gapdh* mRNA expression. The 2^-ΔΔCT^ method was used to calculate relative quantification. (WT vehicle n = 4), (NHERF1 KO vehicle n = 4), (WT cisplatin n = 4), and (NHERF1 KO cisplatin n = 4).

### 2.8. Protein Determination and Immunoblots

Kidney cortex homogenates were made as previously described [19]. Protein concentration was measured by the bicinchoninic acid (BCA) method (Sigma) using BSA as a standard. Kidney homogenates were separated by 10% SDS-PAGE and transferred to nitrocellulose membranes. The membranes were incubated with 5% (wt/vol) dried milk in a Tris-buffered saline with 0.5% Tween 20 (TTBS) for 30 min to decrease nonspecific antibody binding. Membranes were incubated overnight at 4 °C with primary antibodies against GGT (ABclonal; A1776) and CCBL1 (ABclonal; A6542), diluted (1:1000) in 5% milk/TTBS. Membranes were washed with TTBS and incubated for 2 h with horseradish peroxidase-conjugated secondary antibodies diluted in TTBS and 5% milk. Chemiluminescence (ThermoScientific) was utilized to detect bands and visualization via GeneSys software with a Pixi imaging system (Syngene). A densitometric analysis was performed via Image J. For the densitometric analysis, specific protein expression was normalized to densitometry values for glyceraldehyde 3-phosphate dehydrogenase (GAPDH) (Cell Signaling; Beverly, MA, USA) or β-actin (Cell Signaling; Beverly, MA, USA) in each lane.

### 2.9. Inductively Coupled Plasma-Mass Spectrometry of Platinum

Between 15–30 mg of cisplatin treated kidney cortex were snap frozen and stored at −80 °C for ICP-MS analysis. These kidneys were sent to the University of Cincinnati’s Chemistry Department where the samples were processed and analyzed by ICP-MS for platinum (Pt) levels at 24 h: (WT cisplatin: n = 6) and (NHERF1 KO cisplatin: n = 6) and 72 h: (WT cisplatin: n = 8) and (NHERF1 KO cisplatin: n = 8).

### 2.10. Statistical Analysis

A two-way analysis of variance (ANOVA) was used to evaluate changes in GGT activity, changes in body weights of mice, GGT and CCBL in treated kidney cortex by Western blot, reduction/oxidation state of GSH and Cys in plasma and treated kidneys, mRNA expression via qPCR, and urine NGAL from treated mice. A Student’s T-test was used to evaluate Gclc mRNA expression post-hoc and Pt levels between cisplatin treated WT and NHERF1 KO kidneys. *p*-values of <0.05 were considered statistically significant and data were shown as mean ± SEM.

## 3. Results

### 3.1. Kidneys of NHERF1 KO Mice Treated with Cisplatin Show Significantly Greater Damage when Compared to Those from Cisplatin Treated WT Mice at 24 h

**Figure 2:**
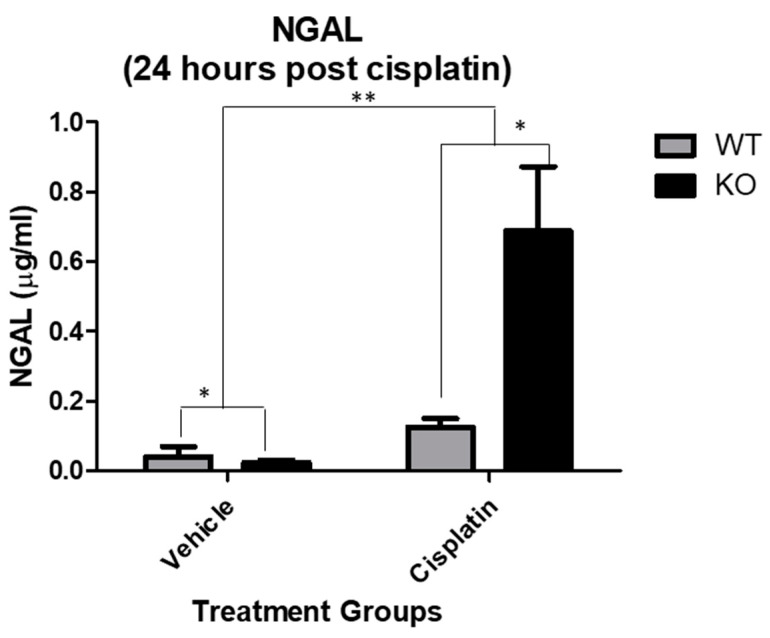
Effect of cisplatin on urine NGAL. NGAL protein measurement of mouse urine in 24 h cisplatin treated WT and NHERF1 KO mouse kidneys. Data are means ± SEM (WT vehicle: n = 5), (NHERF1 KO vehicle: n = 5), (WT cisplatin: n = 7), and (NHERF1 KO cisplatin: n = 8). ** *p* = 0.007 cisplatin treated WT and NHERF1 KO mice compared to vehicle saline controls; * *p* = 0.03 cisplatin treated NHERF1 KO compared to cisplatin treated WT mice; * *p* = 0.04 vehicle treated NHERF1 KO mice compared to vehicle treated WT mice.

We have previously demonstrated significant differences in kidney injury between NHERF1 KO and WT mice 72 h after cisplatin administration [11]. Kidney uptake of cisplatin begins immediately after drug administration, followed by a decrease in tissue levels and a subsequent re-accumulation at 48–72 h. In order to determine if the increased susceptibility to cisplatin-induced nephrotoxicity in NHERF1 KO kidneys manifested during the early phase of cisplatin accumulation, urine NGAL was measured 24 h after administration. Cisplatin increased NGAL protein significantly in both WT (0.1 μg/mL ± 0.02) and NHERF1 KO (0.7 μg/mL ± 0.2) mice compared to vehicle treated WT (0.04 μg/mL ± 0.03) and NHERF1 KO (0.02 μg/mL ± 0.006) (*p* = 0.007) (Figure 2). The increase in the NGAL urine protein level in cisplatin treated NHERF1 KO was significantly greater than cisplatin treated WT mice (*p* = 0.03) (Figure 2).

### 3.2. Greater Renal Histologic Injury 24 h Post Cisplatin in NHERF1 KO Kidneys

**Figure 3:**
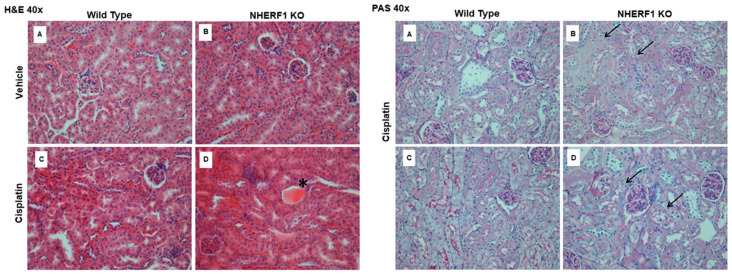
Early histologic effect of cisplatin on WT and NHERF1 KO kidneys. Representative photomicrographs (40×) of H&E (left panel) and PAS staining (right panel) of 24 h-cisplatin treated WT and NHERF1 KO mice. H&E panel (**A**) represents a vehicle treated WT kidney, (**B**) a vehicle treated NHERF1 KO kidney, (**C**) a cisplatin treated WT kidney, and (**D**) a cisplatin treated NHERF1 KO kidney. PAS panel (**A**,**C**) represent cisplatin treated WT kidneys and panel (**B**,**D**) represent cisplatin treated NHERF1 KO kidneys. Asterisks indicate proximal tubule casts and arrows indicate areas BBM sloughing.

H&E and PAS staining of the kidneys of cisplatin treated mice after 24 h revealed that NHERF1 KO mice exhibited a greater degree of early histologic injury when compared to WT mice at the same time point (Figure 3). Similar to what was previously noted at 72 h [11], the injury is predominantly cortical, characterized by casts, atrophy, and BBM sloughing (Figure 3) in cisplatin treated NHERF1 KO mice. Cisplatin treated WT mouse kidneys showed no demonstrable damage. 

### 3.3. NHERF1 KO Kidneys Exhibit Altered Redox

GSH plays a major role in the maintenance of a normal redox state in kidney cells through its antioxidant capacity [20,21]. Cisplatin nephrotoxicity depletes GSH, inducing oxidative stress, which contributes to cellular injury and death. We therefore measured glutathione metabolites by HPLC, including the reduced and oxidized forms of small molecular weight thiols, GSH, GSSG, Cys and CySS, and the mixed disulfide between the two, cysteine-glutathione disulfide (CySSG), on plasma and kidney cortex 72 h after vehicle or cisplatin treatment. 

**Figure 4:**
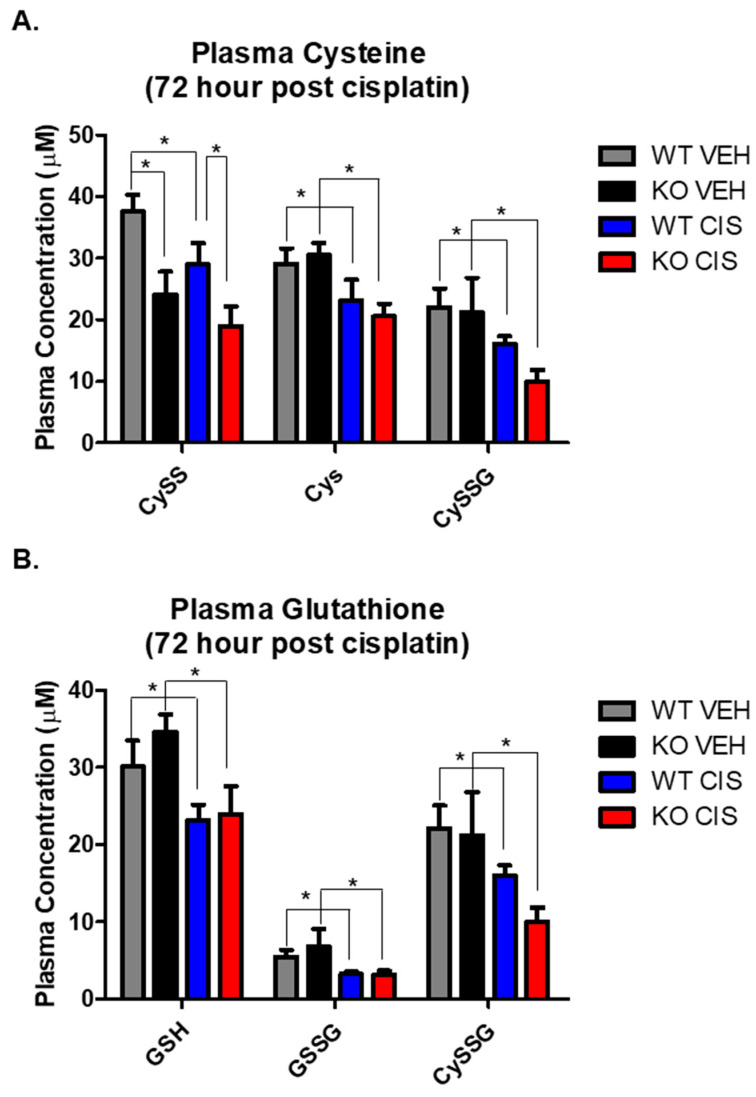

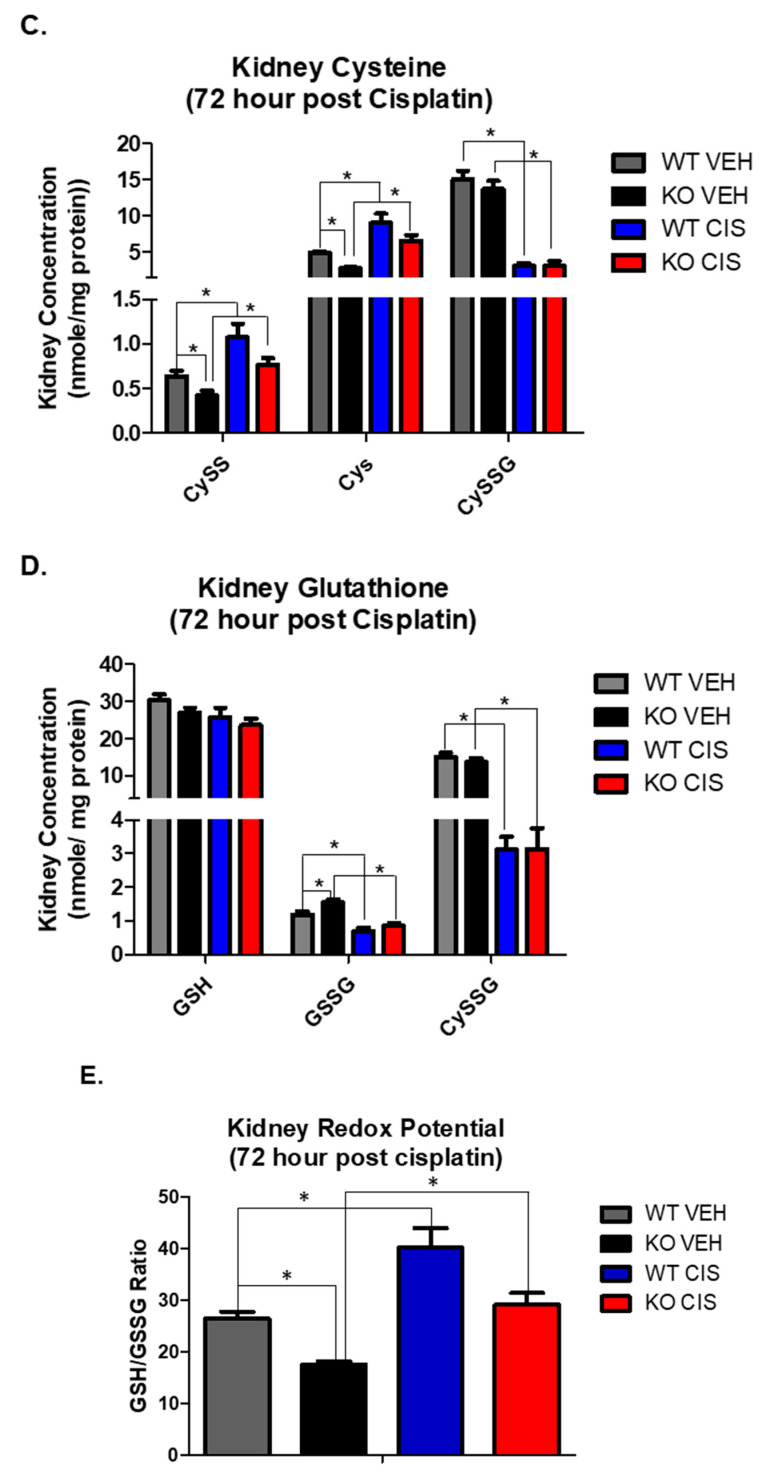
Cisplatin and NHERF1 loss effect on small molecular weight thiols. Plasma and kidney GSG, GSSG, CySSG, Cys, and CySS were measured in 72 h vehicle and cisplatin treated WT and NHERF1 KO mice. (**A**,**B**) Plasma cysteine and glutathione analysis, (**C**,**D**) Kidney cysteine and glutathione analysis, and (**E**) Kidney redox potential analysis. Data are means ± SEM. Plasma: (WT vehicle: n = 5), (NHERF1 KO vehicle: n = 5), (WT cisplatin: n = 7), and (NHERF1 KO cisplatin: n = 9) and kidney: (WT vehicle: n = 5), (NHERF1 KO vehicle: n = 5), (WT cisplatin: n = 8), and (NHERF1 KO cisplatin: n = 9). Plasma: * *p* < 0.05 cisplatin treated WT and NHERF1 KO mice compared to vehicle saline controls for CySS, Cys, CySSG, GSH, GSSG, and CySSG; * *p* < 0.05 vehicle treated NHERF1 KO compared to vehicle treated WT mice for CySS. Kidney: * *p* < 0.05 vehicle treated NHERF1 KO mice compared to vehicle treated WT mice for CySS, Cys, and GSSG; * *p* < 0.05 cisplatin treated WT and NHERF1 KO mice compared to vehicle saline controls for CySS, Cys, CySSG, and GSSG.

In vehicle treated mice, only CySS was significantly decreased in NHERF1 KO plasma relative to WT plasma (*p* = 0.0042) (Figure 4A). In response to cisplatin, most small molecular weight thiols in the plasma were significantly decreased when compared to their respective vehicle controls (Cys *p* = 0.013), (CySSG *p* = 0.003), (GSH *p* = 0.02), and (GSSG *p* = 0.002) (Figure 4A,B). However, only the changes in CySS resulted in a significant difference between cisplatin treated WT and NHERF1 KO animals. 

In the kidney, vehicle treated NHERF1 KO mice showed significantly lower CySS (*p* = 0.027) and Cys levels (*p* = 0.028) and a higher GSSG level (*p* = 0.022) relative to vehicle treated WT mice (Figure 4C,D). Cisplatin treatment led to a significant increase in CySS (*p* = 0.002) and Cys (*p* = 0.0005) and a significant decrease in CySSG (*p* < 0.0001) and GSSG in both genotypes (*p* < 0.0001) (Figure 4C,D). On the other hand, kidney levels of GSH were similar between vehicle treated WT and NHERF1 KO animals and were not affected by cisplatin (Figure 4D). The ratio of reduced to oxidized glutathione (GSH/GSSG), a measure of kidney redox potential, was significantly lower in NHERF1 KO kidneys compared to WT (*p* = 0.003) and increased in both genotypes with cisplatin treatment (*p* = 0.0002) (Figure 4E). The GSH/GSSG ratio consistently remained lower in the cisplatin treated NHERF1 KO kidney in comparison to the cisplatin treated WT kidney.

### 3.4. Comparison of Genes Involved in Glutathione Synthesis between NHERF1 Deficient and WT Kidneys

**Figure 5:**
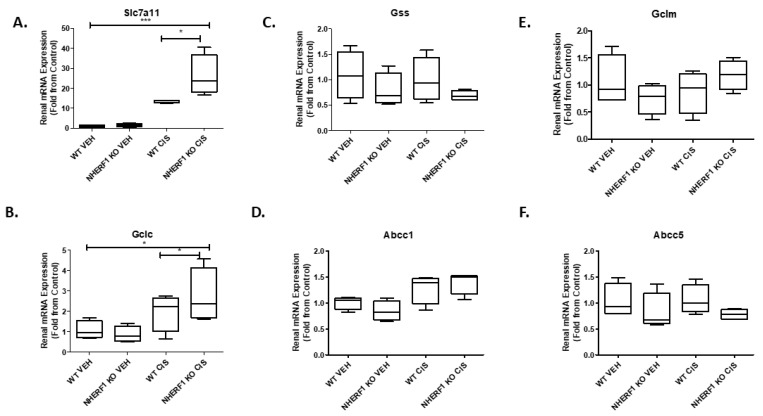
Effect of NHERF 1 loss on mRNA expression of genes involved in GSH synthesis (**A**–**F**). RNA isolated from vehicle and cisplatin treated kidneys was utilized to measure mRNA expression by qPCR analysis as described in the Materials and Methods section. (**A**) qPCR analysis of Slc7a11, (**B**) qPCR analysis of Gclc, (**C**) qPCR analysis of Gss, (**D**) qPCR analysis of Abcc1, (**E**) qPCR analysis of Abcc5. Data are means ± SEM. (WT vehicle: n = 4), (NHERF1 KO vehicle: n = 4), (WT cisplatin: n = 4), and (NHERF1 KO cisplatin: n = 4). *** *p* < 0.0001 *Slc7a11* cisplatin treatment compared to vehicle treatment. * *p* = 0.03 *Slc7a11* cisplatin treated NHERF1 KO compared to cisplatin treated WT. * *p* = 0.0147 *Gclc* cisplatin treatment compared to vehicle treatment. * *p* = 0.035 *Gclc* cisplatin treated NHERF1 KO compared to cisplatin treated WT.

The GSH synthesis is mainly regulated by cellular concentrations of cysteine and the activity of enzymes such as glutamate-cysteine ligase (GCL) [22]. To investigate if there is an effect on glutathione synthesis with NHERF1 loss and 72 h cisplatin treatment, six genes were measured: cystine/glutamate antiporter (*Slc7a11*), glutamate-cysteine ligase catalytic subunit (*Gclc*), glutamate-cysteine ligase modifier (*Gclm*), glutathione synthetase (*Gss*), glutathione-s-conjugate-translocating ATPase (*Abcc1*), and ATP binding cassette subfamily C member 5 (*Abcc5*). Cisplatin significantly increased *Slc7a11* expression in both genotypes (*p* < 0.0001) but to a greater extent in the NHERF1 KO kidneys (*p* = 0.03) (Figure 5A). *Gclc* expression was significantly increased with cisplatin treatment in both genotypes (*p* = 0.0147); however, the expression of *Gclc* was increased in the cisplatin treated NHERF1 KO kidney to a greater degree than cisplatin treated WT (*p* = 0.035) (Figure 5B). The expression levels of *Gss*, *Abcc1*, *Gclm*, and *Abcc5* were not affected regardless of genotype or treatment group (Figure 5C–F). 

### 3.5. Cisplatin-Induced Changes in GGT Activity in WT and KO Kidneys

**Figure 6:**
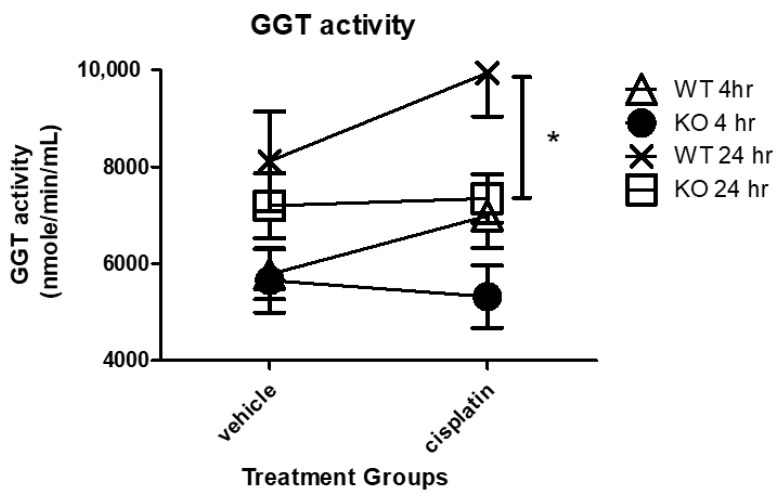
Effect of NHERF1 on GGT activity following cisplatin treatment. GGT activity was measured at 4 h and 24 h after cisplatin treatment in WT and NHERF1 KO kidney cortex homogenates as described in the Materials and Methods section. Data are means ± SEM. 4 h: (WT vehicle: n = 6), (NHERF1 KO vehicle: n = 6), (WT cisplatin: n = 6), and (NHERF1 KO cisplatin: n = 6) and 24 h: (WT vehicle: n = 5), (NHERF1 KO vehicle: n = 5), (WT cisplatin: n = 7), and (NHERF1 KO cisplatin: n = 8). * *p* = 0.04 cisplatin treated NHERF1 KO compared to cisplatin treated WT.

GGT activity, which is responsible for the intracellular recycling of Cys for the synthesis of GSH, plays a major role in the kidney metabolism of cisplatin. Human studies have shown urine GGT activity is at its highest peak 24 h or earlier following cisplatin treatment and then steadily declines [23]. To examine the effect of NHERF1 loss on the time dependent GGT activity after cisplatin, 4 h and 24 h vehicle or cisplatin treated WT and NHERF1 KO kidney GGT activity was measured. GGT activity was similar in WT and NHERF1 KO vehicle treated kidneys at both time points (Figure 6). At 4 h after cisplatin treatment, WT and KO kidneys showed similar GGT activity that was not significantly affected by cisplatin (Figure 6). At 24 h after cisplatin treatment, WT kidneys showed a significantly increased GGT activity relative to NHERF1 KO kidneys [24 h (WT cisplatin: 9900 nmole/min/mL ± 2400), (NHERF1 KO cisplatin: 7300 nmole/min/mL ± 1400)] (*p* = 0.04) (Figure 6). 

### 3.6. Cisplatin Treatment and NHERF1 Loss Affect GGT Protein Expression

**Figure 7:**
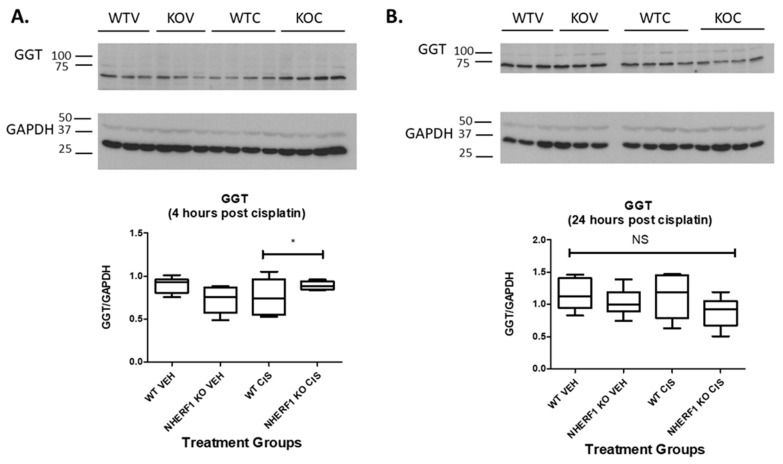
Effect of NHERF1 loss on GGT protein expression. Representative blots of 4 h (**A**) and 24 h (**B**) GGT and GAPDH are shown. Kidney cortex homogenates were separated by 10% SDS-PAGE and transferred to nitrocellulose membranes. Quantitation was performed as described in the Methods section. (**B**) Data are means ± SEM (WT vehicle: n = 6), (NHERF1 vehicle: n = 6), (WT cisplatin: n = 6), and (NHERF1 KO cisplatin: n = 6). * *p* = 0.015 4 h cisplatin treated NHERF1 KO mice compared to cisplatin treated WT mice. (**A**) representative blot from two independent experiments for each time point is shown.

To determine if the decreased GGT activity seen in the NHERF1 KO kidneys was due to changes in GGT protein expression, immunoblot for GGT protein was performed at 4 and 24 h post cisplatin or vehicle treatment in WT and NHERF1 KO kidney homogenates (Figure 7). GGT kidney protein expression was equivalent in WT and KO vehicle treated kidneys at both time points [4 h (WT vehicle: 0.9 GGT/GAPDH ± 0.04), (NHERF1 KO vehicle: 0.7 GGT/GAPDH ± 0.06)] and [24 h (WT vehicle: 1.2 GGT/GAPDH ± 0.1), (NHERF1 KO vehicle: 1.0 GGT/GAPDH ± 0.1)] (Figure 7). However, 4 h post cisplatin treatment resulted in a significant interaction between WT and NHERF1 KO kidneys (*p* = 0.015) [4 h (WT cisplatin: 0.8 GGT/GAPDH ± 0.09), (NHERF1 KO cisplatin: 0.9 GGT/GAPDH ± 0.02)] (Figure 7A). The 24 h cisplatin treatment group showed no significant changes between WT and NHERF1 KO kidneys [24 h (WT cisplatin: 1.1 GGT/GAPDH ± 0.1), (NHERF1 KO vehicle: 0.9 GGT/GAPDH ± 0.1)] (Figure 7B).

### 3.7. Differences in GGT Localization in NHERF1 KO and WT Kidneys

**Figure 8:**
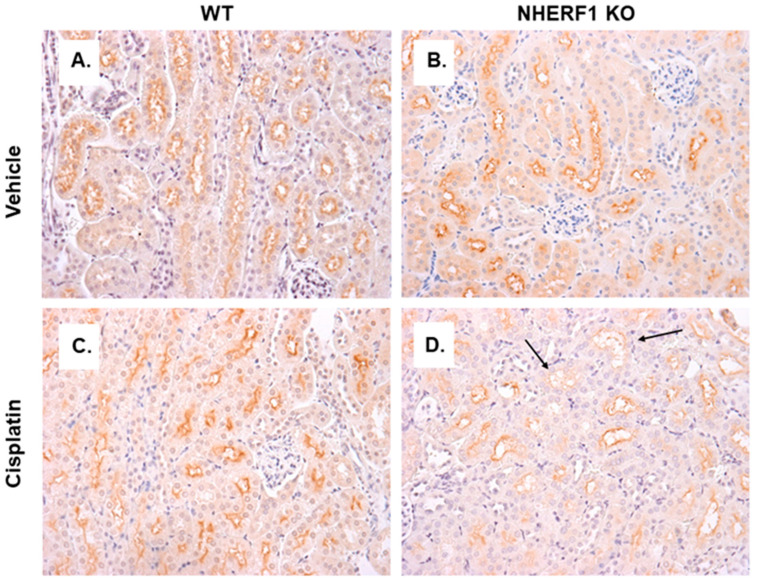
GGT localization in vehicle and cisplatin treated kidneys. Representative photomicrographs (40×) of IHC staining of GGT in 24 h cisplatin treated WT and NHERF1 KO mice. Panel (**A**) represents a vehicle treated WT kidney, (**B**) a vehicle treated NHERF1 KO kidney, (**C**) a cisplatin treated WT kidney, and (**D**) a cisplatin treated NHERF1 KO kidney. Arrows indicate areas of BBM sloughing and GGT staining loss (WT vehicle: n = 4), (NHERF1 KO vehicle: n = 5), (WT cisplatin: n = 4), and (NHERF1 KO: n = 5).

To determine if the differences in GGT activity were reflected in localization of GGT in WT and NHERF1 KO kidneys, an IHC analysis of kidney slices was performed. GGT staining in the proximal tubules of vehicle treated WT and KO kidneys were similar (Figure 8A,B). After cisplatin, WT kidneys’ GGT expression pattern was similar to vehicle treated (Figure 8C). However, the cisplatin treated NHERF1 KO kidneys showed decreased staining for GGT in the BBM compared to cisplatin treated WT or vehicle treated kidneys (Figure 8D). 

### 3.8. NHERF1 Expression Influences CCBL Protein Expression

**Figure 9:**
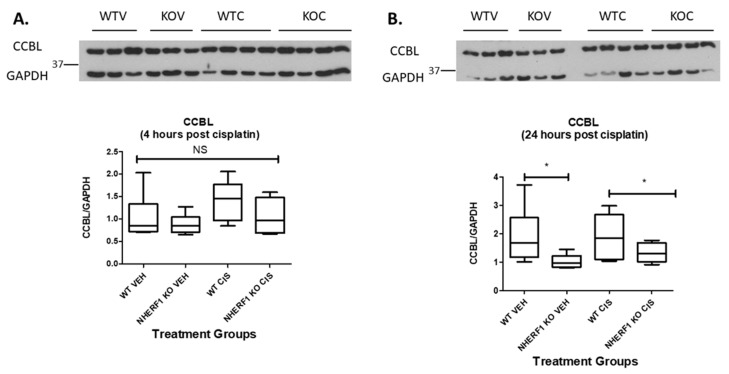
Effect of NHERF1 loss on CCBL protein expression. Representative blots of 4 h (**A**) and 24 h (**B**) CCBL and GAPDH are shown. Kidney cortex homogenates were separated by 10% SDS-PAGE and transferred to nitrocellulose membranes. Quantitation was performed as described in Materials and Methods. (B) Data are means ± SEM [4 h (WT vehicle: n = 6), (NHERF1 vehicle: n = 6), (WT cisplatin: n = 6), and (NHERF1 KO cisplatin: n = 6) and 24 h (WT vehicle: n = 6), (NHERF1 vehicle: n = 6), (WT cisplatin: n = 6), and (NHERF1 KO cisplatin: n = 5). * *p* = 0.018 24 h vehicle treated NHERF1 KO mice compared to vehicle treated WT mice; * *p* = 0.018 24 h cisplatin treated NHERF1 KO mice compared to cisplatin treated WT mice. A representative blot from two independent experiments for each time point is shown.

The expression of CCBL, the pyridoxal phosphate-dependent enzyme involved in intracellular cisplatin metabolism, was also compared by immunoblot in WT and KO mouse kidney homogenates at 4 and 24 h after cisplatin or vehicle therapy. CCBL protein was equivalent between the two genotypes whether vehicle or cisplatin treated at 4 h [4 h (WT vehicle: 1.4 CCBL/GAPDH ± 0.3), (NHERF1 KO vehicle: 1.1 CCBL/GAPDH ± 0.1)] and [4 h (WT cisplatin: 1.7 CCBL/GAPDH ± 0.1), (NHERF1 KO cisplatin: 1.3 CCBL/GAPDH ± 0.2)] (Figure 9A). However, after 24 h there is a genotype affect resulting in a significant decrease in the CCBL protein expression in both vehicle and cisplatin treated NHERF1 KO kidneys (*p* = 0.018) [24 h (WT vehicle: 1.9 CCBL/GAPDH ± 0.4), (NHERF1 KO vehicle: 1.0 CCBL/GAPDH ± 0.1)] and [24 h (WT cisplatin: 1.9 CCBL/GAPDH ± 0.3), (NHERF1 KO cisplatin: 1.3 CCBL/GAPDH ± 0.2)] (*p* = 0.018) (Figure 9B). 

### 3.9. CCBL Localization and Staining Pattern Is Altered in NHERF1 KO Kidneys Prior to Cisplatin Treatment

**Figure 10:**
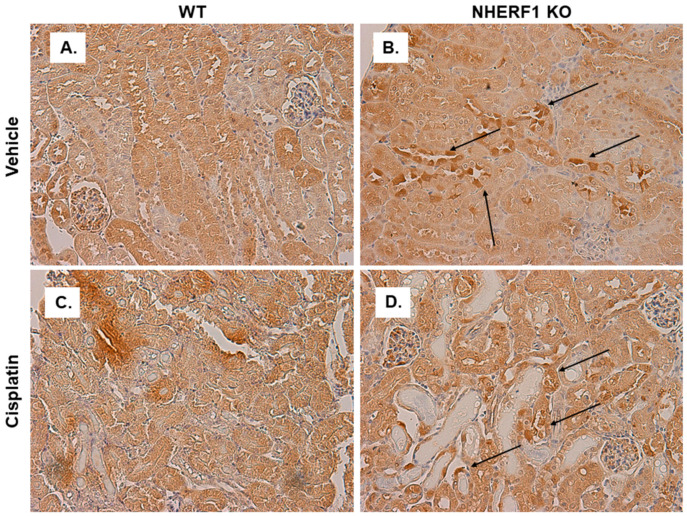
CCBL localization in WT and NHERF1 KO vehicle and cisplatin treated kidneys. Representative photomicrographs (40×) of IHC staining of CCBL1 in 72 h cisplatin treated WT and NHERF1 KO mice. Panel (**A**) represents a vehicle treated WT kidney, (**B**) a vehicle treated NHERF1 KO kidney, (**C**) a cisplatin treated WT kidney, and (**D**) a cisplatin treated NHERF1 KO kidney. Arrows indicate areas of unique staining patterns seen in NHERF1 KO kidneys (WT vehicle: n = 5), (NHERF1 KO vehicle: n = 6), (WT cisplatin: n = 4), and (NHERF1 KO cisplatin: n = 5).

As a scaffolding protein, NHERF1 may play a role in the intracellular localization of enzymes involved in cisplatin metabolism such as CCBL. IHC for CCBL in WT and KO kidney slices after vehicle treatment revealed heterogeneity of cell CCBL staining in the distal and cortical collecting tubules of NHERF1 KO vehicle treated kidneys, even within the same tubule (Figure 10A,B) compared to more homogeneous staining in the WT slices. The NHERF1 KO kidneys appear to maintain their unique staining pattern after treatment with cisplatin (Figure 10D). Following this, we blinded these slides and evaluated: uniform vs. patchy staining, granular staining, heterogeneous tubule mosaic staining, and tubule injury. The distal and cortical collecting tubule heterogeneous mosaic pattern was very distinct in two of the six vehicle treated NHERF1 KO mice, the other four had less noticeable heterogeneous mosaic staining in their tubules. Notably, two of the five cisplatin treated NHERF1 KO kidneys had very distinct tubular granular staining of CCBL, and tubules from these mice had the most injury. 

### 3.10. Pt Levels Are Decreased in NHERF1 KO Kidneys 72 h after Cisplatin Treatment

**Figure 11:**
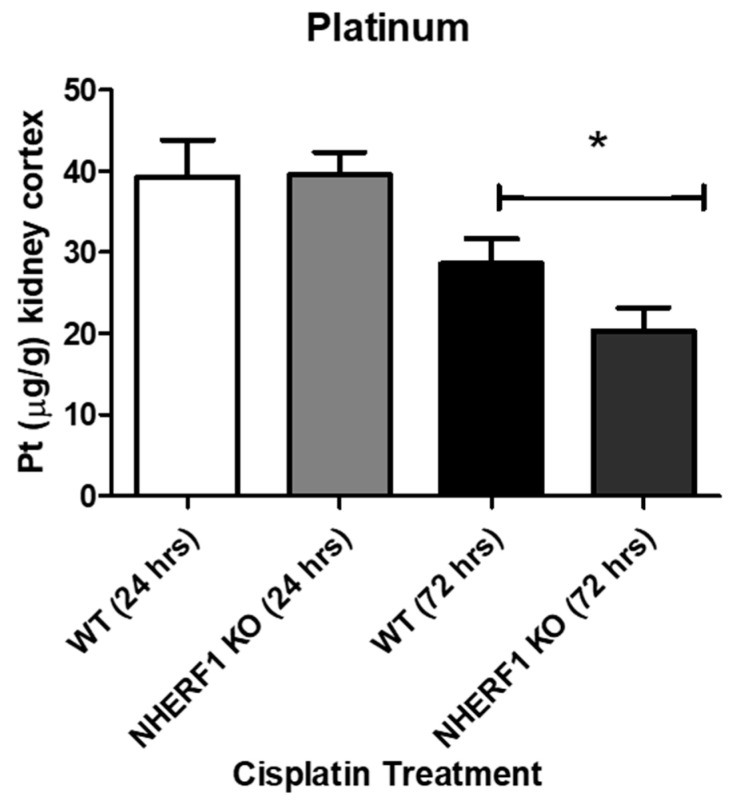
Platinum levels found in 24 and 72 h cisplatin treated WT and NHERF1 kidneys. Pt was measured via ICP-MS at 24 and 72 h post cisplatin treatment in the WT and NHERF1 KO kidney cortex as described in the Methods section. Data are means ± SEM. 24 h: (WT cisplatin: n = 6) and (NHERF1 KO cisplatin: n = 6) and 72 h: (WT cisplatin: n = 8) and (NHERF1 KO cisplatin: n = 8). * *p* = 0.04 cisplatin treated NHERF1 KO COMPARED to cisplatin treated WT.

To evaluate if there were changes in the renal handling of cisplatin via uptake/extrusion, kidneys were harvested for ICP-MS to measure Pt levels. Twenty-four hour cisplatin treated kidneys showed no differences in platinum levels between WT and NHERF1 KO (WT: 39.3 μg/g ± 3.1) (KO: 39.6 μg/g ± 2.8) (*p* = 0.95) (Figure 11). However, at 72 h the NHERF1 KO kidneys (8.0 μg/g ± 2.8) exhibited a significant decrease in Pt content when compared to WT (8.8 μg/g ± 4.6) (*p* = 0.04) (Figure 11). 

## 4. Discussion

The purpose of this study was to address the hypothesis that NHERF1 loss affected the renal handling of cisplatin, as a potential mechanism for the increased susceptibility of NHERF1 KO mice to cisplatin-induced AKI. This hypothesis was based on the principle that the development of cisplatin tubular injury is dependent on cisplatin transport into tubular cells and that NHERF1 affects the expression and function of epithelial transporters [24,25]. This original study to investigate the effect of NHERF1 deficiency on cisplatin transport and metabolism in proximal tubule has produced several key findings, including changes in kidney redox state, altered activity and localization of enzymes involved in cisplatin metabolism, and differential Pt handling in NHERF1 KO mouse kidneys. These provocative findings suggest potential mechanisms for increased susceptibility to cisplatin nephrotoxicity in NHERF1 KO mice and potential targets for prevention of cisplatin injury.

NHERF1 KO mice exhibit greater sensitivity to cisplatin-induced nephrotoxicity at the early time point of 24 h when compared to WT, as manifested in histology and urine NGAL excretion. These data are consistent with the increased sensitivity to cisplatin seen in the previous 72 h study [11] and suggest that the kidneys of the NHERF1 KO animals may be ‘primed’ to sustain greater injury. We hypothesized that an increase in susceptibility to cisplatin nephrotoxicity may stem from the presence of a pre-existing stress state, altered metabolism of cisplatin resulting in increased production or retention of toxic metabolites, or increased exposure due to changes in cisplatin uptake or extrusion. Our results confirm that NHERF1 KO kidneys show greater oxidative stress and changes in enzymes involved in cisplatin metabolism; however, there is no evidence that NHERF1 loss results in greater cisplatin exposure.

NHERF1 KO animals demonstrated changes in the levels of glutathione metabolites under baseline conditions, disclosing the existence of underlying oxidative stress. Previous studies have documented that cisplatin produces alterations in glutathione metabolites, confirmed by our findings that plasma from 72 h cisplatin treated mice showed a decrease in GSH, GSSG, CySSG, Cys, and CySS for both WT and NHERF1 KO when compared to vehicle treated [8,26,27,28]. However, even in the vehicle treated animals NHERF1 loss was accompanied by a decrease in plasma and kidney CySS, the oxidized form of Cys. The importance of this observation is that CySS is the metabolite which is imported into cells and reduced to Cys, which is a necessary substrate for the cellular antioxidant GSH [29]. Coinciding with the decrease in plasma and kidney CySS levels in the NHERF1 KO kidneys, GSSG, the oxidized form of GSH was increased in the kidney of vehicle treated NHERF1 KO mice. Kidney levels of GSH in vehicle treated WT and NHERF1 KO mice were similar, suggesting the possibility that kidney cell uptake of CySS from the plasma is increased, leading to a decrease in the plasma level, in order to maintain equivalent production of GSH within the kidney. Our data do not allow us to confirm this hypothesis. Despite decreased CySSG and GSSG in both genotypes with cisplatin treatment, kidney GSH was not decreased in either genotype [27]. The ultimate result of the genotype-specific differences in glutathione metabolism is a decrease in kidney redox potential (GSH/GSSG) in NHERF1 KO mouse kidneys when compared to WT, driven primarily by increased kidney GSSG relative to kidney GSH levels. In cells, significantly increased GSSG levels result in an unbalanced redox system (GSH/GSSG) leading to the production of superoxide and cell death [30,31]. Furthermore, CySS has been shown to be a key regulator of GSH homeostasis, where suppressed extracellular CySS levels lead to decreased intracellular GSH [29]. We have previously documented an increase in enzymes involved in the pentose phosphate pathway in NHERF1 KO kidneys, which act as a source of NADPH [14]. GSSG is continuously reduced back to GSH through the reaction of NADPH-dependent glutathione disulfide reductase [31,32]; therefore, an increase in enzymes in the pentose phosphate pathway may be a compensatory reaction to increase necessary NADPH to reduce GSSG back to GSH and restore the cellular redox state. Direct measurement of glutathione peroxidase, glutathione synthase, superoxide dismutase, or catalase in WT and NHERF1 KO mice may be useful in providing further insight into the mechanism of susceptibility of NHERF1 KO mice to cisplatin nephrotoxicity. 

Gene expression of *Gclm*, *Gss*, *Abcc1*, and *Abcc5* were not significantly altered with NHERF1 loss or cisplatin treatment. However, *Slc7a11* gene expression was significantly increased with cisplatin treatment in both WT and NHERF1 KO kidneys and to a greater extent in cisplatin treated NHERF1 KO kidneys. *Slc7a11* plays a key role in antioxidant defense through its mediation of CySS uptake which promotes GSH synthesis and the maintenance of cell survival under oxidative stress [33]. Recently, a study published in Nature Cell Biology has shown an increase in *Slc7a11* mediated CySS uptake is important in maintaining redox balance, but it comes at a considerable cost for cancer cells [34]. By actively importing insoluble CySS there is a potential toxic effect; thus, cancer cells with elevated *Slc7a11* must rapidly reduce insoluble CySS to soluble Cys to prevent toxicity. Rapidly reducing insoluble CySS to soluble Cys requires a substantial supply of NADPH from the pentose phosphate pathway [33,34] and results in a significant drain in cellular NADPH. This increase in *Slc7a11* gene expression supports our previous finding of increased enzyme activity within the pentose phosphate pathway, which we previously proposed could be a compensatory mechanism to increase NADPH production due to oxidative stress. Thus, the increase in *Slc7a11* gene expression may be acting in concert with the increase in NADPH production to counteract an increase in oxidative stress and may account for the significantly decreased CySS in both NHERF1 KO plasma and kidneys under baseline conditions. *Gclc* was significantly increased in the cisplatin treated NHERF1 KO kidneys. It is a key player in regulating cellular Cys concentrations and GSH synthesis. Insufficient GSH results in oxidative stress and upregulation of *Gclc* gene expression [22]. The high levels of GSSG and the altered redox potential (GSH/GSSG) in the NHERF1 KO kidneys may explain the upregulation in both *Slc7a11* and *Gclc* gene expression with the additional ‘stress’ from cisplatin. Overall, these data are consistent with previous results where NHERF1 KO kidneys appear to function normally under baseline conditions, but when stressed with cisplatin the kidneys are unable to mount an appropriate response to increased oxidative stress demands.

We also found changes in two of the enzymes, GGT and CCBL, involved in cisplatin metabolism, suggesting an additional mechanism for enhanced cisplatin nephrotoxicity in the NHERF1 KO animals. A robust increase in GGT activity was seen in the 24 h cisplatin treated WT mice, while an increase in GGT activity was not seen in the NHERF1 KO mice, suggesting that NHERF1 is important for GGT’s response to an insult. This finding is somewhat surprising as several studies have indicated that GGT activity and/or expression plays a role in the formation of nephrotoxic cisplatin metabolites [8,35,36]. The role of GGT activity or expression in protection or susceptibility to cisplatin nephrotoxicity is controversial [9,37,38]. GGT KO mice are protected from cisplatin nephrotoxicity [39]. On the other hand, in vitro studies have produced contradictory results. Depending on the cell type, an increase in GGT activity was protective and a decrease in GGT activity enhanced sensitivity to cisplatin toxicity [9,39]. However, the addition of GGT to rescue these cells did not result in protection but instead increased cisplatin sensitivity [9,39]. These data have led to two alternative hypotheses: (1) GGT metabolizes cisplatin to a nephrotoxin, thus inhibition is protective, or (2) GGT detoxifies xenobiotics and produces GSH, thus alleviating oxidative stress from cisplatin [9,27,39,40]. The second theory may explain the exacerbated AKI in NHERF1 KO mice. If NHERF1 loss limits the ability of GGT to respond quickly and/or robustly to an insult there may be increased injury. It is notable that despite a modest increase in GGT expression at 4 h after cisplatin treatment in the NHERF1 KO animals compared to WT, this did not translate into increased activity. This observation suggests that NHERF1 loss plays a role in the integrity of the signaling and/or metabolic pathway leading to activation of GGT. Whether this is a direct effect of NHERF1 or an indirect effect cannot be answered from these data. Note that at 24 h after cisplatin, there was a mild but nonsignificant decrease in GGT expression. IHC showed greater GGT loss in the proximal tubule of NHERF1 KO kidneys possibly due to BBM sloughing. This GGT loss has been recorded in mice treated with cisplatin for 72 h in addition to a decrease in GGT activity at 72 h [23,41,42]. These findings suggest that, in addition to the early defect in cisplatin-induced GGT activity, the failure of GGT activity to increase at the later time points in NHERF1 KO kidneys may be the result of more severe early damage and loss of BBM and consequently GGT resulting in exaggerated injury after the initial insult. In other words, early injury begets later injury. Clearly, more research is needed to understand the role GGT plays in the renal response to cisplatin.

CCBL is the rate limiting enzyme in the metabolism of cisplatin to the ‘reactive thiol’ that has been implicated in nephrotoxicity [8,35,36,43], hence the importance of identifying any effect of NHERF1 loss on CCBL protein level, localization, or activity. In this work, CCBL protein level was investigated and found to be similar regardless of genotype or treatment at 4 h and significantly decreased in NHERF1 KO kidneys when compared to WT at 24 h. IHC evaluating CCBL localization revealed a heterogenous staining in many tubules of NHERF1 KO animals. In two of the five cisplatin treated NHERF1 KO mice, there was a very distinct granular staining of CCBL in the tubules. Notably, these animals also had the most injury compared to others in the NHERF1 KO group and to WT kidneys. This staining pattern may indicate alterations in CCBL activity or perhaps shifts in CCBL processing in the NHERF1 KO kidneys. An altered CCBL activity could affect the degree of injury and suggest additional pathways for the exacerbated injury in the kidneys of NHERF1 KO mice. Future experiments will also evaluate CCBL activity between vehicle and cisplatin treated WT and NHERF1 KO mice kidneys. 

Lastly, Pt levels in cisplatin treated WT and NHERF1 KO kidneys were investigated using ICP-MS. We hypothesized that altered transport of cisplatin in NHERF1 KO kidneys, particularly decreased activity of MATE1, the extrusion transport protein in the apical membrane of proximal tubule cells, would result in higher Pt levels and increased toxicity from cisplatin [6,13,41]. Interestingly, no change in Pt levels was seen 24 h post treatment, suggesting that the uptake transporter, OCT2, is expressed and functional in NHERF1 KO kidneys. However, at 72 h cisplatin treated NHERF1 KO kidneys showed a significant decrease in Pt levels when compared to their respective controls, suggesting that extrusion of cisplatin from the proximal tubule cell is not blocked. Alternatively, the decrease in Pt levels at 72 h in the NHERF1 KO kidneys compared to WT may be due to increased necrosis in the proximal tubule where Pt is being lost as the membrane integrity of the tubule decreases.

**Figure 12:**
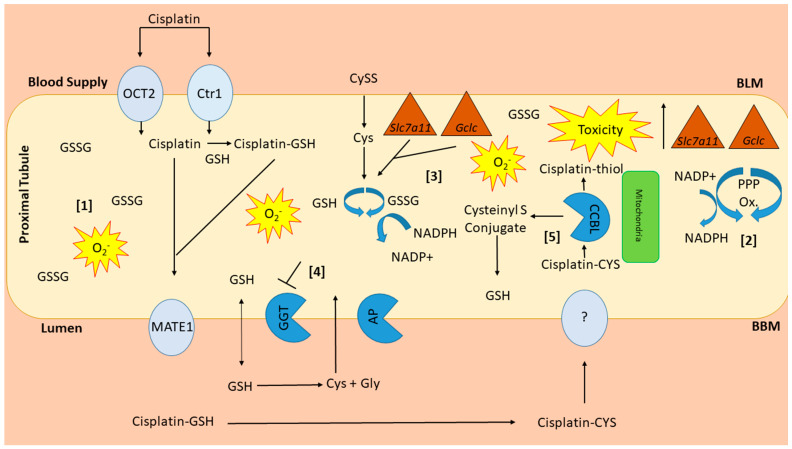
Working hypothesis of susceptibility of NHERF1 KO kidneys to cisplatin nephrotoxicity. NHERF1 KO mouse kidneys have increased levels of GSSG [1], a known pro-oxidant. To help alleviate the oxidative stress NHERF1 KO kidneys have increased enzymes from the pentose phosphate pathway (G6PDH & ME) [2] to produce more NADPH, needed for the conversion of GSSG to GSH [3]. Additional stress from cisplatin results in increases in gene expression of *Slc7a11* and *Gclc* which are increased to regulate cellular levels of Cys and increase GSH production [3]. GGT, an enzyme important for the recycling of cysteine and GSH, is unable to respond to the increased demand for more GSH in response to the oxidizing conditions [4]. The introduction of cisplatin decreases G6PDH and ME activity resulting in a decrease in NADPH, reducing its ability to convert GSSG to GSH and alleviate this excess oxidative stress. Lastly, an increased activity of CCBL [5] would also increase the amount of the cisplatin-thiol resulting in more toxicity.

Ultimately, there are still unanswered questions regarding the mechanism of cisplatin nephrotoxicity; however, it is evident that NHERF1 expression plays a vital role in its development. These data have provided more clarity regarding the underlying mechanism of susceptibility to cisplatin in NHERF1 KO mice. Furthermore, future experiments (e.g., CCBL activity, measurement of kidney tissue NAD+/NADPH, and antioxidant enzyme activity) are integral in providing further insight into the mechanism of susceptibility. These data in combination with our previous discovery of an altered pentose phosphate pathway in NHERF1 KO kidneys [14] have led to the development of a working hypothesis regarding the mechanism of susceptibility to cisplatin nephrotoxicity (Figure 12). 

NHERF1 KO mouse kidneys have increased levels of GSSG, a known pro-oxidant [30,31] (Figure 12). Elevated levels of GSSG can lead to an unbalanced cellular redox state [30,31]. In an effort to restore the cellular redox state NHERF1 KO kidneys have increased enzymes of the pentose phosphate pathway (Glucose 6-Phosphate Dehydrogenase (G6PDH) & Malic Enzyme (ME)) which results in increased production of NADPH, needed to convert GSSG to GSH [14,32] (Figure 12). Following cisplatin administration *Slc7a11* and *Gclc* gene expression are increased to enhance cellular uptake of Cys and increase GSH production, expanding the demand for NADPH. Lastly, GGT, an essential enzyme involved in the recycling of Cys for the de novo synthesis of GSH appears to be unable to respond to the increased demand for GSH in response to cisplatin (Figure 12). Studies in cancer cells have shown inhibition of GGT results in a shift to more oxidizing conditions and sensitizes these cells to oxidative stress induced by chemotherapies [44,45,46,47]. Cisplatin treatment decreases G6PDH and ME activity, which would be expected to exacerbate the oxidative stress induced by cisplatin by limiting NADPH production needed to convert GSSG back to GSH (Figure 12). NHERF1 KO kidneys, already more dependent on NADPH produced through the pentose phosphate pathway than WT kidneys, would be more susceptible to the effects of cisplatin. These three conditions in the NHERF1 KO kidneys–decreased redox potential, increased reliance on the pentose phosphate pathway for NADPH production, and inability to respond to cisplatin with an increase in GGT activity—are all likely to contribute to the exacerbated nephrotoxic response to cisplatin. 

## 5. Conclusions

In summary, we have demonstrated unique mechanisms for susceptibility to cisplatin-induced AKI related to the scaffolding protein, NHERF1. NHERF1 deficient kidneys exhibit evidence of underlying oxidative stress manifested by a higher level of oxidized glutathione and altered metabolic response to cisplatin manifested by the lack of increase in GGT activity and altered CCBL localization. The former condition likely predisposes to greater injury inflicted by the profound oxidative insult of cisplatin, while the latter defects may contribute by preventing appropriate metabolic handling of the drug. Which of these plays the more important role in enhancing the AKI injury cannot be determined from this study, but the findings highlight further avenues for investigation. Coupled with our prior studies showing increased activity of the pentose phosphate pathway in NHERF1 deficient kidneys, these findings add to a growing body of knowledge implicating NHERF1 in the regulation of cell metabolism. Interestingly, and despite the fact that NHERF1 is best recognized as a scaffolding protein for epithelial transporters, the NHERF1 deficient kidneys show equivalent uptake and even enhanced expulsion of cisplatin, suggesting that altered cisplatin transport does not play a role in this phenomenon. Translation of these findings into the clinical realm may lead to better ways to prevent cisplatin-induced AKI, allowing clinicians to better apply this powerful chemotherapeutic agent.

## Data Availability

All dates in this paper.

## Supplementary Materials

The following are available online at https://www.mdpi.com/article/10.3390/antiox10071036/s1, Figure S1. Body weights of mice were recorded prior to cisplatin/vehicle administration (Day 0) and 72 h after cisplatin administration (Day 3).
