# Supplementary material for: Na/H Exchange Regulatory Factor 1 Deficient Mice Show Evidence of Oxidative Stress and Altered Cisplatin Pharmacokinetics
Source: Antioxidants (Basel). 2021 Jun 28;10(7):1036. doi: 10.3390/antiox10071036 (PMC8300832; doi:10.3390/antiox10071036)
Supplement: Supplementary file 1 [file antioxidants-10-01036-s001.zip › antioxidants-1250334-supplementary.pdf]

Supplementary Materials: Figure S1. Body weights of mice were recorded prior to cisplatin/vehicle administration (Day 0) and 72 h after cisplatin administration (Day 3).

Body weights of mice were recorded prior to cisplatin or vehicle administration (Day 0) and at the time of sacrifice (Day 3) (Figure S1). There were no significant changes in body weight between vehicle treated WT and NHERF1 KO mice on Day 0 or Day 3 (Figure S1). After cisplatin administration (Day 3) both WT and NHERF1 KO mice exhibited similar decreases in body weight, and no significant differences in body weight were found between cisplatin treated WT and NHERF1 KO mice on Day 3 (Figure S1).

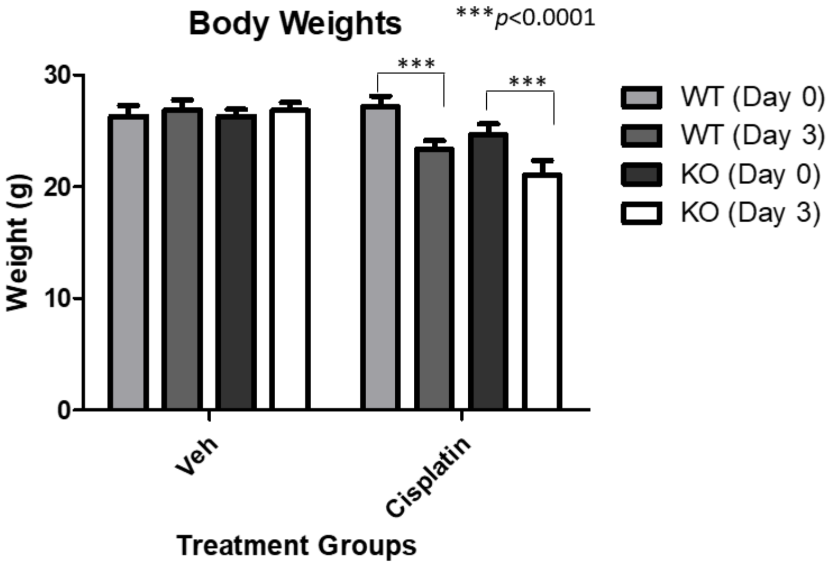

Figure S1. Body weights of mice were recorded prior to cisplatin/vehicle administration (Day 0) and 72 h after cisplatin administration (Day 3). Data are means ± SEM. (WT vehicle: n = 5), (NHERF1 KO vehicle: n = 5), (WT cisplatin: n = 8), and (NHERF1 KO cisplatin: n = 9). *** $p < 0.0001$ Day 3 cisplatin treated WT compared to Day 0 cisplatin treated WT. *** $p < 0.0001$ Day 3 cisplatin treated NHERF1 KO compared to Day 0 cisplatin treated NHERF1 KO.
